# The effect of positioning and diaphragmatic breathing exercises on respiratory muscle activity in people with chronic obstructive pulmonary disease

**DOI:** 10.4102/sajp.v72i1.315

**Published:** 2016-06-29

**Authors:** Brenda Morrow, Jarred Brink, Samantha Grace, Lisa Pritchard, Alison Lupton-Smith

**Affiliations:** 1 Department of Paediatrics and Child Health, University of Cape Town, Cape Town, South Africa; 2 Department of Health and Rehabilitation Sciences, University of Cape Town, Cape Town, South Africa

## Abstract

**Background:**

Body positioning and diaphragmatic breathing may alter respiratory pattern and reduce dyspnoea in people with chronic obstructive pulmonary disease (COPD).

**Objectives:**

To determine the effect of positioning and diaphragmatic breathing on respiratory muscle activity in a convenience sample of people with COPD, using surface electromyography (sEMG).

**Methods:**

This prospective descriptive study recorded sEMG measurements at baseline, after upright positioning, during diaphragmatic breathing and 5 minutes thereafter. Vital signs and levels of perceived dyspnoea were recorded at baseline and at the end of the study. Data were analysed using repeated measures ANOVAs with post hoc *t*-tests for dependent and independent variables.

**Results:**

Eighteen participants (13 male; mean ± standard deviation age 59.0 ± 7.9 years) were enrolled. Total diaphragmatic activity did not change with repositioning (*p* = 0.2), but activity increased from 7.3 ± 4.2 µV at baseline to 10.0 ± 3.3 µV during diaphragmatic breathing (*p* = 0.006) with a subsequent reduction from baseline to 6.1 ± 3.5 µV (*p* = 0.007) at the final measurement. There was no change in intercostal muscle activity at different time points (*p* = 0.8). No adverse events occurred. Nutritional status significantly affected diaphragmatic activity (*p* = 0.004), with participants with normal body mass index (BMI) showing the greatest response to both positioning and diaphragmatic breathing. There were no significant changes in vital signs, except for a reduction in systolic/diastolic blood pressure from 139.6 ± 18.7/80.4 ± 13.0 to 126.0 ± 15.1/75.2 ± 14.7 (*p* < 0.05).

**Conclusion:**

A single session of diaphragmatic breathing transiently improved diaphragmatic muscle activity, with no associated reduction in dyspnoea.

## Introduction

Chronic obstructive pulmonary disease (COPD) is a common disease that is both treatable and preventable (Global Initiative for Chronic Obstructive Lung Disease 2015). It is characterised by progressive airflow limitation and hyperinflation, associated with shortness of breath or dyspnoea and altered respiratory patterns, which become progressively worse and are the main cause of morbidity and mortality globally (Varga 2015). The prevalence of COPD in South Africa is high from a global perspective, at > 19%, attributed to the significant burden of tuberculosis, smoking and occupational dust exposure (Abdool-Gaffar *et al*. 2011).

Body positioning and breathing techniques are common physiotherapy techniques used to relieve dyspnoea (Gosselink 2003; 2004; Mikelsons 2008), despite limited supporting evidence (Cahalin *et al*. 2002; Holland *et al*. 2012). Diaphragmatic breathing is one breathing technique, which aims to reduce dyspnoea by increasing diaphragmatic excursion and simultaneously reducing accessory muscle use (which contributes greatly to work of breathing) and correcting abnormal chest wall movement (Cahalin *et al*. 2002; Fernandes, Cukier & Feltrim 2011; Yamaguti *et al*. 2012).

Some reports have shown diaphragmatic breathing to cause a significant increase in tidal volume, reduction in respiratory rate, and improved breathing pattern and respiratory efficiency in COPD patients (Fernandes *et al*. 2011; Yamaguti *et al*. 2012). An example of a COPD patient’s perception of the utility of diaphragmatic breathing was, ‘Diaphragmatic breathing has been extremely beneficial to my ability to function in daily life and to the quality of my personal, recreational, and professional life’ (Sharma 2005). However, there is also a concern that in patients with severe COPD and asynchronous thoraco-abdominal motion, diaphragmatic breathing may actually cause an increase in dyspnoea and reduce the mechanical efficiency of breathing (Fernandes *et al*. 2011; Gosselink *et al*. 1995; Mikelsons 2008; Vitacca *et al*. 1998). Therefore, it is important to determine whether the effect of diaphragmatic breathing differs amongst participants with varying disease severity.

The use of diaphragmatic breathing in people with COPD remains controversial, but continues to be used in physiotherapy practice. Some studies of diaphragmatic breathing techniques have measured abdominal expansion to determine the effect on diaphragmatic function (Gosselink *et al*. 1995; Yamaguti *et al*. 2012), but it is unclear whether abdominal movement is specific to diaphragmatic muscle activity – it is quite possible to expand the abdomen with minimal or no diaphragmatic involvement (Sharma 2005). Direct measurement of diaphragmatic muscle activity may therefore be a better outcome measure. The effect of nutritional status on the effects of positioning and diaphragmatic breathing is not known, but it is postulated that increased body mass index (BMI), which may be associated with increased abdominal adipose tissue deposition, could impact negatively on diaphragm activity and the potential to recruit diaphragmatic activity during diaphragmatic breathing.

This study aimed to examine the short-term effect of posture correction and positioning and diaphragmatic breathing on respiratory muscle activity (diaphragm and intercostal muscles) in patients with COPD, using surface electromyography (sEMG). In addition, we aimed to assess the short-term effect of positioning and diaphragmatic breathing on perceived dyspnoea and haemodynamic status, and whether diaphragmatic muscle activity response to the study interventions was affected by nutritional status or disease severity measured by COPD ‘GOLD’ classification (Table 1).

**TABLE 1:** ‘GOLD’ classification of COPD severity (Global Initiative for Chronic Obstructive Lung Disease 2015).

Stage	Characteristics
1: Mild COPD	FEV1/FVC < 70%
	FEV1 > or equal to 80% predicted
	With or without chronic symptoms (cough, sputum production)
2: Moderate COPD	FEV1/FVC < 70%
	FEV1 between 50 and 80% predicted
	With or without chronic symptoms (cough, sputum production)
3: Severe COPD	FEV1/FVC < 70%
	FEV1 between 30 and 50% predicted
	With or without chronic symptoms (cough, sputum production)a
4: Very severe COPD	FEV1/FVC < 70%
	FEV1 < or equal to 30% predicted or FEV1 < 50% predicted plus chronic respiratory failure

*Source*: Authors’ own work

## Methods

### Research design and participants

This was a prospective observational study, using a convenience sample of spontaneously breathing participants with COPD and forced expiratory volume in one second (FEV1) less than 80% predicted (Global Initiative for Chronic Obstructive Lung Disease, GOLD, stage ≥ 2), recruited from Groote Schuur Hospital, Cape Town, South Africa. Institutional Human Research Ethics Committee approval was obtained for this study (HREC 073/2015) and informed consent taken from all participants before enrolling.

Participants with active implants (e.g. pacemakers and cochlear implants); coexisting lung pathology; neuromuscular disease; or a history of pulmonary embolus, cardiovascular instability or any other life-threatening conditions were excluded from the study.

### Sample size

Considering that most published studies using sEMG in adults enrolled < 15 participants, we planned *a priori* to conduct a sample size analysis using data collected after enrolling 15 participants. Using collected data at this point, the final required sample size was determined to be *n* = 18 participants in order to detect a mean difference in total diaphragmatic activity of 2 µV with a standard deviation of 2.5 µV (alpha 0.05, power 0.8).

### Procedure

Participants were asked to position themselves in their most comfortable position, if not positioned as such already. Each participant’s baseline level of perceived dyspnoea was assessed using a Modified Borg Dyspnoea Scale, as described by Kendrick, Baxi and Smith (2000), and baseline vital signs (transcutaneous oxygen saturation respiratory rate, heart rate, and blood pressure (BP)) were recorded. The modified Borg Scale has previously been shown to be valid and reliable in measuring perceived dyspnoea in people with COPD (Kendrick *et al*. 2000).

A Dipha^®^ sEMG device (Inbiolab BV, Groningen, the Netherlands) was used to measure the muscle activity of the diaphragm and intercostal muscles. sEMG has previously been shown to be a valid and reliable method for assessing respiratory muscle activity (Duiverman *et al*. 2004; Maarsingh *et al*. 2000). sEMG electrodes were placed in a standardised arrangement on the participants’ thorax, as previously described (Maarsingh *et al*. 2000). sEMG measurements were then recorded for two minutes in the participants’ position of choice (measurement 1, baseline). The participant’s posture was then corrected and position changed to an upright, supported seated position, with the arms resting comfortably forward or at the sides (but not weight-bearing as in the tripod position). If the participant was unable to sit upright, for any reason, they were positioned in a semi-Fowler’s position with the head of the bed raised between 30 and 45 degrees. A further two-minute sEMG recording was taken in this position (measurement 2) after allowing the participant to recover from any exertion during the position change.

The participant was then taught diaphragmatic breathing by the same person in a standardised fashion, facilitated by tactile stimulation, as previously described (Cahalin *et al*. 2002). Once the participant was able to perform the technique satisfactorily, as indicated by visible abdominal excursion (Cahalin *et al*. 2002; Fernandes *et al*. 2011), sEMG readings were recorded for a further two minutes whilst performing diaphragmatic breathing (measurement 3). The participant was then allowed to rest for five minutes again in their chosen position of comfort, after which the participant’s vital signs, Borg Scale and sEMG measurements were again recorded (measurement 4, final).

Data were processed and analysed offline using Polybench software (Inbiolab BV, Groningen, the Netherlands) and then exported for further analysis. The average muscle activity over each measurement period was used for analysis, and data for the dorsal and ventral diaphragmatic components were pooled (total diaphragmatic activity). To improve rigour, the data were coded so that the analyst was blinded to sequence.

### Statistical analysis

Data were tested for normality using the Shapiro–Wilks W test. Descriptive data are presented as mean ± standard deviation or 95% confidence interval and proportions (*n*%) as appropriate to normally distributed data. One-way and two-way repeated measures (between and within groups) ANOVAs were used to assess changes in sEMG activity in individual muscle groups (diaphragm and intercostal) between the four measurement points of the study. Post hoc *t*-tests for dependent variables were conducted to determine between which time points significant changes occurred; and post hoc *t*-tests for independent variables were done to determine significant differences at specific time points between groups. Statistica (version 12 StatSoft Inc., Tulsa, USA) was used for statistical analysis. A significance level of *p* < 0.05 was chosen.

## Results

Eighteen participants (13 male) were enrolled in the study (Table 2). Complete nutritional data could not be obtained for one participant. All participants completed the study interventions, and there were no adverse events.

**TABLE 2:** Baseline participant characteristics (*n* = 18).

Characteristics	Results
**Age (years)**	59.0 ± 7.9
**Gender (male: female)**	13: 5
**Smoking history (pack years)**	29.8 ± 16.0
**GOLD classification**	
Stage 2	6 (33.3%)
Stage 3	11 (61.1%)
Stage 4	1 (5.6%)
Body mass index (kg/m^2^) (*n* = 17)	25.5 ± 7.8
**Nutritional status based on BMI (*n* = 17)**	
Normal	7 (41.2%)
Underweight	3 (17.6%)
Pre-obese	4 (23.5%)
Obese	3 (17.6%)
**Comorbid conditions (*n* = 12, 66.7%)**	
Hypertension	11 (91.7%)
Diabetes mellitus	6 (50%)
Systemic lupus erythematosus	1 (8.3%)
Chronic renal disease	1 (8.3%)
Epilepsy (controlled)	1 (8.3%)
Osteoarthritis	1 (8.3%)
Hypothyroidism	1 (8.3%)

*Source*: Authors’ own work

Comorbidities, most commonly hypertension, were common, occurring in 12 (66.7%) participants (some had more than one comorbid condition) (Table 2). Five (27.8%) participants were current smokers.

Four (22.2%) participants were receiving oxygen therapy: one via nasal prongs and three via facemask with FiO_2_ = 0.4. Participants all chose a slouched position of comfort, with a kyphotic thorax, protracted shoulders and a forward poking chin, either in supported sitting (*n* = 13) or low semi-Fowlers (*n* = 5). None assumed a tripod position. After posture correction and positioning, subsequent measurements were taken in upright sitting (*n* = 16) or high semi-Fowlers (*n* = 2).

**FIGURE 1:**
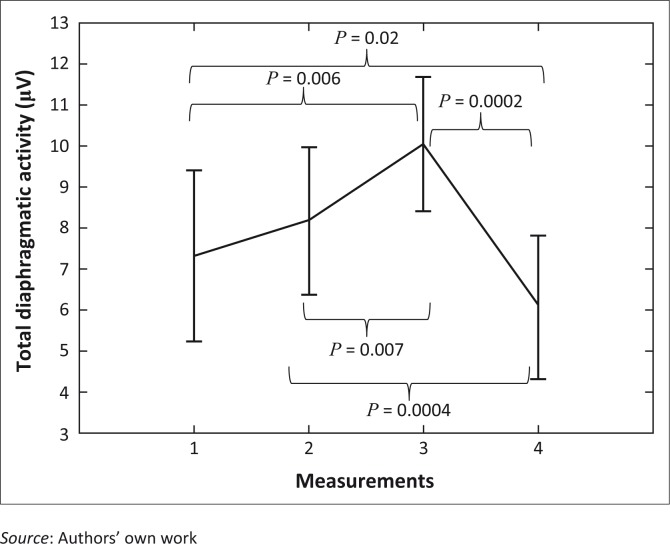
Mean change in total diaphragmatic activity at different measurement points (sequence). Vertical bars denote 95% confidence intervals, overall effect *p* < 0.0001. Significant changes between measurement points are noted.

sEMG readings for total diaphragmatic and intercostal muscle activity at different measurement points are presented in Table 3. There was no significant change in intercostal muscle activity (*p* = 0.8) across the study period, but there was a significant change in total diaphragmatic activity at different measurement points (*p* < 0.0001, Figure 1). The difference in total diaphragmatic activity between participants’ initial position of choice (measurement 1) and the corrected position (measurement 2) was not significant (*p* = 0.2); however, total diaphragmatic activity increased significantly from baseline (measurement 1) to during diaphragmatic breathing (measurement 3, *p* = 0.006) and between correcting the participants’ positions (measurement 2) and during diaphragmatic breathing (measurement 3, *p* = 0.0004) (Figure 1). There was also a significant reduction in total diaphragmatic activity (*p* = 0.007) between baseline and the final measurement (4), both in the participants’ chosen positions of comfort (Figure 1).

**TABLE 3:** Surface electromyographic readings at different measurement points.

Variable	Measurement			

	1	2	3	4
Total diaphragmatic activity (µV)	7.3 ± 4.2	8.2 ± 3.6	10.0 ± 3.3	6.1 ± 3.5
Intercostal muscle activity (µV)	4.1 ± 2.8	4.0 ± 4.0	5.3 ± 5.3	3.6 ± 2.9

*Source*: Authors’ own work

**FIGURE 2:**
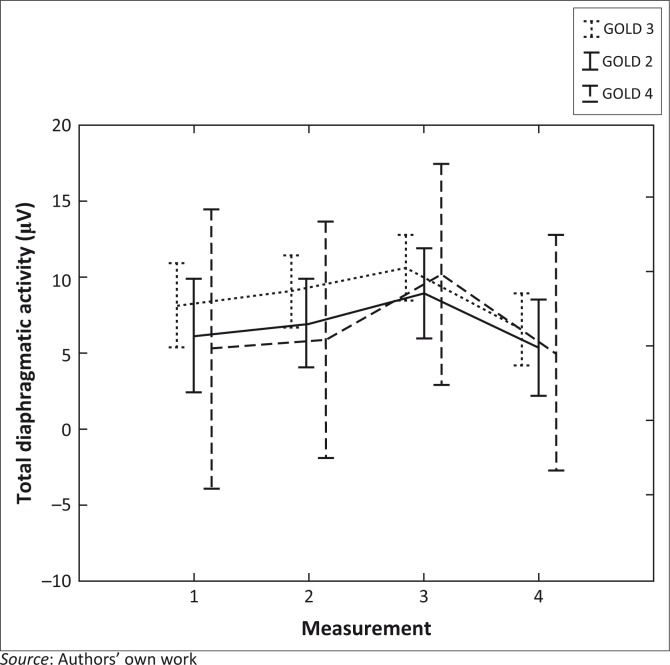
Change in diaphragmatic muscle activity according to GOLD classification. Points are mean and vertical bars denote 95% confidence interval, overall effect *p* = 0.96.

There was no association between GOLD classification and change in total diaphragmatic activity over the study period (*p* = 0.96, Figure 2).

**FIGURE 3:**
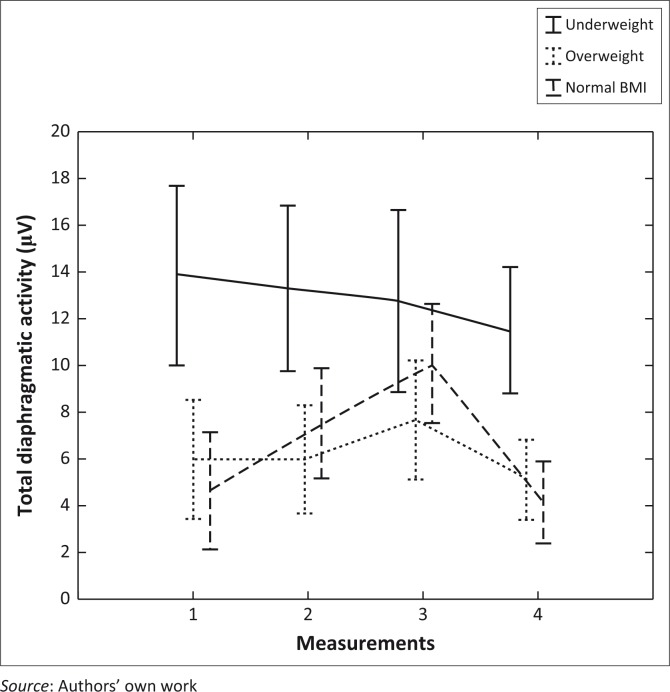
Effect of nutritional status on change in total diaphragmatic activity. Points are mean and vertical bars denote 95% confidence interval, overall effect *p* = 0.004.

Nutritional status had a significant effect on diaphragmatic activity (*p* = 0.004, Figure 3), with underweight participants (according to BMI) showing the highest diaphragmatic activity with the least response to positioning and diaphragmatic breathing whilst patients with normal BMI showed the greatest response to both positioning and diaphragmatic breathing.

Participants classified as underweight had significantly higher diaphragmatic activity than those classified as overweight at all measurement points, and this was also significantly higher than participants with normal BMI at measurements 1,2 and 4 (*p* < 0.05) (Figure 3).

Participants with normal BMI showed increased diaphragmatic activity between baseline (measurement 1) and measurement 2 (*p* = 0.04) and between measurements 1 and 3 (*p* = 0.03), with a subsequent decrease in diaphragmatic activity between measurements 3 and 4 (*p* = 0.0007) (Figure 3). Participants classified as overweight by BMI had an increase in diaphragmatic activity between measurements 2 and 3 (*p* = 0.03), with no other significant changes. Participants classified as underweight by BMI had no significant change in diaphragmatic activity throughout the study period.

There was a significant decrease in systolic and diastolic BP following the intervention (*p* < 0.05, Table 4), but no change in other haemodynamic parameters or in the Borg Scale of perceived dyspnoea (Table 4).

**TABLE 4:** Changes in secondary outcome measures between baseline (measurement 1) and the final measurement (measurement 4).

Variable	Measurement 1	Measurement 4	*P*-value
Modified Borg Dyspnoea Scale	5.4 ± 2.9	4.8 ± 2.8	0.1
Transcutaneous oxygen saturation (%)	96.1 ± 2.4	96.2 ± 2.2	0.7
Heart rate (beats per minute)	89.6 ± 18.8	87.5 ± 19.5	0.5
Respiratory rate (breaths per minute)	21.6 ± 5.5	20.2 ± 6.1	0.2
Systolic blood pressure (mmHg)	139.6 ± 18.7	126.0 ± 15.1	0.003
Diastolic blood pressure (mmHg)	80.4 ± 13.0	75.2 ± 14.7	0.03

*Source*: Authors’ own work

## Discussion

To the best of our knowledge, this is the first study demonstrating the effects of position change and diaphragmatic breathing on diaphragmatic and intercostal muscle activity in adult patients with COPD.

We found no significant response to posture correction and position change in either diaphragmatic or intercostal muscle activity. We chose to position participants in either upright supported sitting or high semi-Fowlers, as generally recommended (Cahalin *et al*. 2002). We avoided the tripod position in order to minimise accessory muscle use (pectoralis minor and major), as it has been suggested that the forward leaning tripod position with arm or head support enables these muscles to significantly contribute to rib cage elevation (Gosselink 2004). However, considering that the forward leaning position has also been shown to improve diaphragmatic function and reduce dyspnoea, the effect of diaphragmatic breathing in this position warrants investigation (Gosselink 2004).

It is worth noting that although many people with COPD have been reported to adopt the tripod position, none of the participants in our study chose this as their position of comfort, instead opting for a slouched, kyphotic position which may have impeded diaphragmatic function by abdominal compression. Our findings are in line with a previous report that diaphragmatic movements were not significantly affected by position change (sitting, lying supine and tripod position) in people with stable COPD (Bhatt *et al*. 2009). We did not assess the position of the diaphragm and degree of hyperinflation through imaging, and this should be considered for future studies.

During diaphragmatic breathing, diaphragmatic activity increased significantly, with no change in intercostal muscle activity. This suggests that diaphragmatic breathing was taught and implemented correctly, without recruiting the intercostal muscles. Previous studies have also observed increased abdominal motion following diaphragmatic breathing (Gosselink *et al*. 1995; Vitacca *et al*. 1998; Yamaguti *et al*. 2012), which may reflect increased diaphragmatic activity. Five minutes after completing diaphragmatic breathing, however, diaphragmatic activity was significantly reduced from baseline, with no positive carry-over effect of the intervention. Although this study only measured the short-term effects of a single treatment intervention, other studies have investigated the effects of longer diaphragmatic breathing training programmes in patients with COPD (Fernandes *et al*. 2011; Gosselink *et al*. 1995; Yamaguti *et al*. 2012). Yamaguti *et al*. (2012) showed a significant improvement in abdominal motion and diaphragmatic mobility following a four-week diaphragmatic breathing training programme (Yamaguti *et al*. 2012). These findings, together with our findings of increased diaphragm muscle activity after a single diaphragmatic breathing session, highlight the potential for clinical benefits if diaphragmatic breathing is taught over a longer time frame. This requires further investigation in longer term studies. We conducted the final sEMG measurement in the participant’s position of comfort, as it is usual for patients to reassume their position of comfort after a therapy session. Future studies should evaluate the relative carry-over of diaphragmatic breathing effect with patients remaining in an upright, well-aligned body position.

No significant change in participants’ perceptions of dyspnoea following diaphragmatic breathing was reported. Previous reports have been conflicting, with some observing increased levels of dyspnoea (Gosselink *et al*. 1995) and others a decrease in dyspnoea after diaphragmatic breathing (Cahalin *et al*. 2002; Yamaguti *et al*. 2012). Possible explanations for this discrepancy include the subjective nature of the Borg Scale, potential problems in understanding the scale elements, and differences in implementation and training of diaphragmatic breathing (Cahalin *et al*. 2002). A pictorial Borg Scale could be considered for future studies to improve participant understanding.

BP measurements decreased significantly following the study interventions, and this has not been previously reported. The explanation for this observation is unclear, but may be attributed to increased relaxation, although similar effects were not observed in any other secondary outcome measure.

There was no difference in respiratory muscle activity response to study interventions between participants with different COPD severity, using GOLD criteria (Table 1). This was assessed as it has been suggested that the response to diaphragmatic breathing may differ according to severity of disease (Cahalin *et al*. 2002; Fernandes *et al*. 2011; Mikelsons 2008). Our results are unable to explain these reported differences on the basis of diaphragmatic activity.

Nutritional status of participants had a significant effect on diaphragmatic muscle activity. An overweight BMI classification is likely to be associated with greater abdominal adipose tissue, thus increasing the resistance against which the diaphragm must work to achieve diaphragmatic breathing. However, increased adipose tissue may have influenced the sEMG readings, because of increased distance from the muscles themselves. This may explain the apparent reduced response to diaphragmatic breathing in these participants compared to those classified as having normal BMI. Participants classified as being underweight had greater diaphragmatic muscle activity than other groups. Underweight people are more likely to have a reduction in diaphragm muscle mass (Dureuil & Matuszczak 1998), but muscle mass may not directly relate to electrical muscle activity. Participants with low BMI did not respond positively to the diaphragmatic breathing or positioning interventions, possibly due to the lack of inhibiting abdominal bulk, allowing optimal diaphragmatic function with no need for correction. It is therefore unsurprising that participants with normally classified BMI showed the greatest diaphragmatic activity response to positioning and diaphragmatic breathing.

### Recommendations

Diaphragmatic breathing clearly improves diaphragmatic muscle activity; however, we cannot determine whether this effect is associated with clinical benefit on the basis of this study. The question of clinical benefit of diaphragmatic breathing in COPD has been raised previously (Cahalin *et al*. 2002; Gosselink *et al*. 1995; Vitacca *et al*. 1998) and requires further investigation. There is currently insufficient evidence to support this treatment technique for reducing dyspnoea in people with COPD.

### Limitations

This study was limited by the relatively small, but adequately powered, sample size and the fact that it was conducted in a single centre. We only assessed the short-term physiological effects of a single session of diaphragmatic breathing, therefore cannot determine the clinical utility of this technique. We only included stable patients with COPD, and these results cannot therefore be generalised to other population groups, including unstable patients with COPD and acute pulmonary exacerbations. The lack of control group and non-randomised participant selection also constitute limitations of this study.

## Conclusions

This study has shown that, in people with COPD GOLD Stage 2 or higher, postural correction and upright positioning had no impact on respiratory muscle activity, whilst diaphragmatic breathing resulted in a transient increase in diaphragmatic activity, with no change in intercostal muscle activity. Perception of dyspnoea was not affected by the study interventions. Whilst there was no difference in diaphragmatic activity response for participants with different GOLD classifications, nutritional status did significantly affect diaphragmatic activity response to positioning and diaphragmatic breathing, with the greatest response occurring in those with normal BMI. Vital signs remained constant, except for a reduction in BP following study interventions.
